# Supplementary material for: Incidence of pelvic fractures after definitive radiotherapy for cervical cancer: A retrospective multicenter cohort study (The IPFAR study)
Source: PLoS One. 2026 Mar 27;21(3):e0344384. doi: 10.1371/journal.pone.0344384 (PMC13028549; doi:10.1371/journal.pone.0344384)
Supplement: S1 Data — (DOCX) [file pone.0344384.s001.docx]

Institution

menopause BMI FIGOstage

PIF Pain

RT field

Time from the end of RT to the onset of PIF (months)

| A | Postmenopausal | under 23 | 4b | Yes | None | Whole pelvis | 16 |
| --- | --- | --- | --- | --- | --- | --- | --- |
| A | Postmenopausal | over 23 | 3a | Yes | Yes | Whole pelvis | 13 |
| A | Postmenopausal | over 23 | 2b | Yes | None | WP+PAN | 5 |
| A | Postmenopausal | under 23 | 2b | Yes | None | WP+PAN | 13 |
| A | Postmenopausal | under 23 | 1b2 | Yes | Yes | WP+PAN | 18 |
| A | Premenopausal | over 23 | 3b | None | None | WP+HDR-ICBT | 30 |
| A | Premenopausal | over 23 | 2b | None | None | WP+HDR-ICBT | 14 |
| A | Premenopausal | over 23 | 2b | None | None | WP+HDR-ICBT | 63 |
| A | Premenopausal | over 23 | 1b1 | None | None | WP+HDR-ICBT | 66 |
| A | Premenopausal | over 23 | 3b | None | None | WP+HDR-ICBT | 64 |
| A | Premenopausal | over 23 | 3b | None | None | WP+HDR-ICBT | 12 |
| A | Premenopausal | over 23 | 2b | None | None | WP+HDR-ICBT | 51 |
| A | Premenopausal | over 23 | 3b | None | None | WP+HDR-ICBT | 50 |
| A | Premenopausal | over 23 | 2b | None | None | WP+HDR-ICBT | 44 |
| A | Premenopausal | over 23 | 1b2 | None | None | WP+HDR-ICBT | 41 |
| A | Postmenopausal | over 23 | 3c1 | None | None | WP+HDR-ICBT | 67 |
| A | Postmenopausal | over 23 | 2b | None | None | WP+HDR-ICBT | 13 |
| A | Postmenopausal | over 23 | 3b | None | None | WP+HDR-ICBT | 59 |
| A | Postmenopausal | over 23 | 1b2 | None | None | WP+HDR-ICBT | 61 |
| A | Postmenopausal | over 23 | 4b | None | None | WP+HDR-ICBT | 38 |
| A | Postmenopausal | over 23 | 2b | None | None | WP+HDR-ICBT | 15 |
| A | Postmenopausal | over 23 | 1b1 | None | None | WP+HDR-ICBT | 21 |
| A | Postmenopausal | over 23 | 2b | None | None | WP+HDR-ICBT | 51 |
| A | Postmenopausal | over 23 | 2a1 | None | None | WP+HDR-ICBT | 52 |
| A | Postmenopausal | over 23 | 2b | None | None | WP+HDR-ICBT | 3 |
| A | Postmenopausal | over 23 | 3b | None | None | WP+HDR-ICBT | 15 |
| A | Postmenopausal | over 23 | 2b | None | None | WP+HDR-ICBT | 46 |
| A | Postmenopausal | over 23 | 2b | None | None | WP+HDR-ICBT | 28 |
| A | Postmenopausal | over 23 | 3b | None | None | WP+HDR-ICBT | 26 |
| A | Postmenopausal | over 23 | 2b | None | None | WP+HDR-ICBT | 45 |
| A | Postmenopausal | over 23 | 2b | None | None | WP+HDR-ICBT | 27 |
| A | Postmenopausal | over 23 | 2b | None | None | WP+HDR-ICBT | 43 |
| A | Postmenopausal | over 23 | 3b | None | None | WP+HDR-ICBT | 37 |
| A | Premenopausal | under 23 | 1b2 | None | None | WP+HDR-ICBT | 68 |
| A | Premenopausal | under 23 | 2a1 | None | None | WP+HDR-ICBT | 68 |
| A | Premenopausal | under 23 | 2b | None | None | WP+HDR-ICBT | 36 |
| A | Premenopausal | under 23 | 2b | None | None | WP+HDR-ICBT | 62 |
| A | Premenopausal | under 23 | 2b | None | None | WP+HDR-ICBT | 47 |
| A | Premenopausal | under 23 | 1b2 | None | None | WP+HDR-ICBT | 50 |
| A | Premenopausal | under 23 | 2b | None | None | WP+HDR-ICBT | 44 |
| A | Postmenopausal | under 23 | 3b | None | None | WP+HDR-ICBT | 69 |
| A | Postmenopausal | under 23 | 1b1 | None | None | WP+HDR-ICBT | 66 |
| A | Postmenopausal | under 23 | 2b | None | None | WP+HDR-ICBT | 45 |
| A | Postmenopausal | under 23 | 2b | None | None | WP+HDR-ICBT | 11 |
| A | Postmenopausal | under 23 | 2b | None | None | WP+HDR-ICBT | 17 |
| A | Postmenopausal | under 23 | 2b | None | None | WP+HDR-ICBT | 45 |
| A | Postmenopausal | under 23 | 3b | None | None | WP+HDR-ICBT | 45 |
| A | Postmenopausal | under 23 | 2b | None | None | WP+HDR-ICBT | 44 |
| A | Postmenopausal | under 23 | 1b1 | None | None | WP+HDR-ICBT | 39 |
| A | Postmenopausal | under 23 | 1b1 | None | None | WP+HDR-ICBT | 43 |
| A | Postmenopausal | over 23 | 3b | Yes | None | WP+HDR-ICBT | 20 |
| A | Postmenopausal | over 23 | 3b | Yes | None | WP+HDR-ICBT | 7 |
| A | Postmenopausal | over 23 | 2b | Yes | None | WP+HDR-ICBT | 19 |
| A | Postmenopausal | over 23 | 2b | Yes | None | WP+HDR-ICBT | 7 |
| A | Postmenopausal | over 23 | 3b | Yes | None | WP+HDR-ICBT | 9 |
| A | Postmenopausal | over 23 | 2b | Yes | None | WP+HDR-ICBT | 17 |
| A | Postmenopausal | over 23 | 4b | Yes | None | WP+HDR-ICBT | 11 |
| A | Premenopausal | over 23 | 1b2 | None | Yes | WP+HDR-ICBT | 19 |
| A | Premenopausal | over 23 | 2a1 | None | Yes | WP+HDR-ICBT | 50 |
| A | Premenopausal | over 23 | 1b1 | None | Yes | WP+HDR-ICBT | 46 |
| A | Premenopausal | over 23 | 2a2 | None | Yes | WP+HDR-ICBT | 26 |
| A | Postmenopausal | over 23 | 2b | None | Yes | WP+HDR-ICBT | 64 |
| A | Postmenopausal | over 23 | 2a2 | None | Yes | WP+HDR-ICBT | 51 |
| A | Postmenopausal | over 23 | 1b1 | None | Yes | WP+HDR-ICBT | 40 |
| A | Postmenopausal | over 23 | 2a2 | None | Yes | WP+HDR-ICBT | 39 |
| A | Premenopausal | under 23 | 1b1 | None | Yes | WP+HDR-ICBT | 69 |
| A | Premenopausal | under 23 | 2b | None | Yes | WP+HDR-ICBT | 50 |
| A | Postmenopausal | under 23 | 1b1 | None | Yes | WP+HDR-ICBT | 71 |
| A | Postmenopausal | under 23 | 3b | None | Yes | WP+HDR-ICBT | 17 |
| A | Postmenopausal | under 23 | 1b2 | None | Yes | WP+HDR-ICBT | 49 |
| A | Premenopausal | over 23 | 3b | Yes | Yes | WP+HDR-ICBT | 2 |
| A | Postmenopausal | over 23 | 2b | Yes | Yes | WP+HDR-ICBT | 11 |
| A | Postmenopausal | under 23 | 2b | Yes | Yes | WP+HDR-ICBT | 27 |
| A | Premenopausal | over 23 | 3b | None | None | WP+PAN+HDR-ICBT | 63 |
| A | Premenopausal | over 23 | 2b | None | None | WP+PAN+HDR-ICBT | 42 |
| A | Postmenopausal | over 23 | 3b | None | None | WP+PAN+HDR-ICBT | 35 |
| A | Postmenopausal | over 23 | 3a | None | None | WP+PAN+HDR-ICBT | 50 |
| A | Premenopausal | under 23 | 3b | None | None | WP+PAN+HDR-ICBT | 46 |
| A | Postmenopausal | under 23 | 3c2 | None | None | WP+PAN+HDR-ICBT | 36 |
| A | Postmenopausal | under 23 | 4a | None | None | WP+PAN+HDR-ICBT | 48 |
| A | Postmenopausal | over 23 | 1b2 | Yes | Yes | WP+PAN+HDR-ICBT | 4 |
| B | Postmenopausal | over 23 | 3b | None | None | Whole pelvis | 29 |
| B | Postmenopausal | over 23 | 4b | None | None | Whole pelvis | 18 |

| B | Postmenopausal | over 23 | 4b | None | None | Whole pelvis | 41 |
| --- | --- | --- | --- | --- | --- | --- | --- |
| B | Postmenopausal | under 23 | 4a | None | None | Whole pelvis | 11 |
| B | Postmenopausal | over 23 | 4b | Yes | None | Whole pelvis | 7 |
| B | Postmenopausal | over 23 | 4a | None | Yes | Whole pelvis | 7 |
| B | Postmenopausal | over 23 | 4a | Yes | Yes | Whole pelvis | 13 |
| B | Postmenopausal | under 23 | 4a | None | None | WP+PAN | 39 |
| B | Premenopausal | over 23 | 2b | None | None | WP+HDR-ICBT | 57 |
| B | Premenopausal | over 23 | 3b | None | None | WP+HDR-ICBT | 60 |
| B | Premenopausal | over 23 | 4b | None | None | WP+HDR-ICBT | 37 |
| B | Premenopausal | over 23 | 2b | None | None | WP+HDR-ICBT | 21 |
| B | Postmenopausal | over 23 | 2b | None | None | WP+HDR-ICBT | 18 |
| B | Postmenopausal | over 23 | 3b | None | None | WP+HDR-ICBT | 57 |
| B | Postmenopausal | over 23 | 3b | None | None | WP+HDR-ICBT | 61 |
| B | Postmenopausal | over 23 | 2b | None | None | WP+HDR-ICBT | 30 |
| B | Postmenopausal | over 23 | 2b | None | None | WP+HDR-ICBT | 53 |
| B | Postmenopausal | over 23 | 3b | None | None | WP+HDR-ICBT | 49 |
| B | Postmenopausal | over 23 | 1b1 | None | None | WP+HDR-ICBT | 46 |
| B | Premenopausal | under 23 | 4a | None | None | WP+HDR-ICBT | 27 |
| B | Premenopausal | under 23 | 2b | None | None | WP+HDR-ICBT | 64 |
| B | Premenopausal | under 23 | 4b | None | None | WP+HDR-ICBT | 8 |
| B | Premenopausal | under 23 | 3c1 | None | None | WP+HDR-ICBT | 60 |
| B | Premenopausal | under 23 | 2b | None | None | WP+HDR-ICBT | 44 |
| B | Premenopausal | under 23 | 3b | None | None | WP+HDR-ICBT | 53 |
| B | Postmenopausal | under 23 | 4b | None | None | WP+HDR-ICBT | 72 |
| B | Postmenopausal | under 23 | 2b | None | None | WP+HDR-ICBT | 68 |
| B | Postmenopausal | under 23 | 3b | None | None | WP+HDR-ICBT | 4 |
| B | Postmenopausal | under 23 | 2b | None | None | WP+HDR-ICBT | 59 |
| B | Postmenopausal | under 23 | 3c1 | None | None | WP+HDR-ICBT | 12 |
| B | Postmenopausal | over 23 | 3b | Yes | None | WP+HDR-ICBT | 20 |
| B | Postmenopausal | over 23 | 2b | Yes | None | WP+HDR-ICBT | 26 |
| B | Postmenopausal | over 23 | 2b | Yes | None | WP+HDR-ICBT | 7 |
| B | Postmenopausal | over 23 | 2b | Yes | None | WP+HDR-ICBT | 24 |
| B | Postmenopausal | over 23 | 3b | Yes | None | WP+HDR-ICBT | 52 |
| B | Postmenopausal | under 23 | 3c1 | Yes | None | WP+HDR-ICBT | 33 |
| B | Postmenopausal | under 23 | 3c1 | Yes | None | WP+HDR-ICBT | 6 |
| B | Postmenopausal | under 23 | 3a | Yes | None | WP+HDR-ICBT | 24 |
| B | Premenopausal | over 23 | 2b | None | Yes | WP+HDR-ICBT | 52 |
| B | Premenopausal | over 23 | 4a | None | Yes | WP+HDR-ICBT | 4 |
| B | Postmenopausal | over 23 | 1b2 | None | Yes | WP+HDR-ICBT | 35 |
| B | Premenopausal | over 23 | 3c1 | Yes | Yes | WP+HDR-ICBT | 13 |
| B | Postmenopausal | over 23 | 3a | Yes | Yes | WP+HDR-ICBT | 12 |
| B | Postmenopausal | over 23 | 3c1 | Yes | Yes | WP+HDR-ICBT | 16 |
| B | Postmenopausal | over 23 | 3c1 | Yes | Yes | WP+HDR-ICBT | 0 |
| B | Postmenopausal | over 23 | 2b | Yes | Yes | WP+HDR-ICBT | 14 |
| B | Postmenopausal | over 23 | 3c1 | Yes | Yes | WP+HDR-ICBT | 24 |
| B | Postmenopausal | under 23 | 1b1 | Yes | Yes | WP+HDR-ICBT | 11 |
| B | Postmenopausal | under 23 | 4a | Yes | Yes | WP+HDR-ICBT | 38 |
| B | Premenopausal | over 23 | 2b | None | None | WP+PAN+HDR-ICBT | 44 |
| B | Premenopausal | over 23 | 3c2 | None | None | WP+PAN+HDR-ICBT | 48 |
| B | Postmenopausal | over 23 | 3b | None | None | WP+PAN+HDR-ICBT | 29 |
| B | Postmenopausal | under 23 | 3c2 | None | None | WP+PAN+HDR-ICBT | 61 |
| B | Postmenopausal | under 23 | 4b | None | None | WP+PAN+HDR-ICBT | 21 |
| B | Premenopausal | under 23 | 3c1 | None | Yes | WP+PAN+HDR-ICBT | 45 |
| B | Postmenopausal | over 23 | 3c2 | Yes | Yes | WP+PAN+HDR-ICBT | 4 |
| C | Premenopausal | over 23 | 4a | None | None | Whole pelvis | 59 |
| C | Postmenopausal | over 23 | 4a | None | None | Whole pelvis | 75 |
| C | Postmenopausal | over 23 | 4a | None | None | Whole pelvis | 18 |
| C | Postmenopausal | over 23 | 3c2 | None | None | Whole pelvis | 0 |
| C | Postmenopausal | over 23 | 2b | None | None | Whole pelvis | 43 |
| C | Premenopausal | under 23 | 1b3 | None | None | Whole pelvis | 9 |
| C | Postmenopausal | under 23 | 3c2 | Yes | None | Whole pelvis | 20 |
| C | Premenopausal | over 23 | 4a | None | Yes | Whole pelvis | 8 |
| C | Premenopausal | over 23 | 4b | None | Yes | Whole pelvis | 61 |
| C | Premenopausal | over 23 | 4b | None | None | WP+PAN | 73 |
| C | Premenopausal | over 23 | 3c1 | None | None | WP+HDR-ICBT | 70 |
| C | Premenopausal | over 23 | 2a2 | None | None | WP+HDR-ICBT | 58 |
| C | Premenopausal | over 23 | 1b2 | None | None | WP+HDR-ICBT | 60 |
| C | Premenopausal | over 23 | 3c1 | None | None | WP+HDR-ICBT | 51 |
| C | Postmenopausal | over 23 | 3b | None | None | WP+HDR-ICBT | 78 |
| C | Postmenopausal | over 23 | 3c1 | None | None | WP+HDR-ICBT | 65 |
| C | Postmenopausal | over 23 | 3b | None | None | WP+HDR-ICBT | 35 |
| C | Postmenopausal | over 23 | 2a1 | None | None | WP+HDR-ICBT | 48 |
| C | Postmenopausal | over 23 | 2a1 | None | None | WP+HDR-ICBT | 48 |
| C | Premenopausal | under 23 | 3c1 | None | None | WP+HDR-ICBT | 14 |
| C | Postmenopausal | under 23 | 3b | None | None | WP+HDR-ICBT | 72 |
| C | Postmenopausal | under 23 | 3c1 | None | None | WP+HDR-ICBT | 78 |
| C | Postmenopausal | under 23 | 2b | None | None | WP+HDR-ICBT | 69 |
| C | Postmenopausal | under 23 | 3c1 | None | None | WP+HDR-ICBT | 58 |
| C | Postmenopausal | under 23 | 3c1 | None | None | WP+HDR-ICBT | 16 |
| C | Postmenopausal | under 23 | 3c1 | None | None | WP+HDR-ICBT | 47 |
| C | Postmenopausal | under 23 | 2b | None | None | WP+HDR-ICBT | 40 |
| C | Postmenopausal | under 23 | 3c1 | None | None | WP+HDR-ICBT | 55 |
| C | Postmenopausal | over 23 | 4b | None | Yes | WP+HDR-ICBT | 4 |
| C | Postmenopausal | under 23 | 3c1 | None | Yes | WP+HDR-ICBT | 32 |
| C | Postmenopausal | over 23 | 1b1 | Yes | Yes | WP+HDR-ICBT | 4 |
| C | Postmenopausal | over 23 | 3c1 | Yes | Yes | WP+HDR-ICBT | 28 |
| C | Postmenopausal | over 23 | 1b1 | Yes | Yes | WP+HDR-ICBT | (3) |
| C | Postmenopausal | under 23 | 3c1 | Yes | Yes | WP+HDR-ICBT | 6 |
| C | Postmenopausal | under 23 | 1b2 | Yes | Yes | WP+HDR-ICBT | 24 |
| C | Postmenopausal | under 23 | 3c1 | None | None | WP+PAN+HDR-ICBT | 49 |

| C | Postmenopausal | over 23 | 3c1 | Yes | None | WP+PAN+HDR-ICBT | 5 |
| --- | --- | --- | --- | --- | --- | --- | --- |
| D | Premenopausal | over 23 | 3c1 | None | None | Whole pelvis | 6 |
| D | Postmenopausal | over 23 | 3c1 | None | None | Whole pelvis | 53 |
| D | Postmenopausal | over 23 | 4a | Yes | None | WP+PAN | 25 |
| D | Premenopausal | over 23 | 2b | None | None | WP+HDR-ICBT | 46 |
| D | Premenopausal | over 23 | 1b2 | None | None | WP+HDR-ICBT | 47 |
| D | Postmenopausal | over 23 | 3a | None | None | WP+HDR-ICBT | 62 |
| D | Premenopausal | under 23 | 2a1 | None | None | WP+HDR-ICBT | 77 |
| D | Premenopausal | under 23 | 2b | None | None | WP+HDR-ICBT | 72 |
| D | Premenopausal | under 23 | 2b | None | None | WP+HDR-ICBT | 53 |
| D | Premenopausal | under 23 | 2a2 | None | None | WP+HDR-ICBT | 46 |
| D | Postmenopausal | under 23 | 2b | None | None | WP+HDR-ICBT | 53 |
| D | Postmenopausal | under 23 | 2b | None | None | WP+HDR-ICBT | 20 |
| D | Postmenopausal | over 23 | 2b | Yes | None | WP+HDR-ICBT | 42 |
| D | Postmenopausal | over 23 | 4a | Yes | None | WP+HDR-ICBT | (0) |
| D | Postmenopausal | under 23 | 1b2 | Yes | None | WP+HDR-ICBT | 35 |
| D | Postmenopausal | under 23 | 3c2 | Yes | None | WP+HDR-ICBT | 30 |
| D | Premenopausal | over 23 | 1ｂ1 | Yes | Yes | WP+HDR-ICBT | 16 |
| D | Postmenopausal | over 23 | 2b | Yes | Yes | WP+HDR-ICBT | 24 |
| D | Postmenopausal | over 23 | 3c2 | Yes | Yes | WP+HDR-ICBT | 13 |
| D | Postmenopausal | under 23 | 2b | Yes | Yes | WP+HDR-ICBT | 11 |
| D | Postmenopausal | over 23 | 4a | None | None | WP+PAN+HDR-ICBT | 5 |
| D | Postmenopausal | over 23 | 2b | None | None | WP+PAN+HDR-ICBT | 63 |
| D | Postmenopausal | over 23 | 3c2 | None | None | WP+PAN+HDR-ICBT | 60 |
| D | Postmenopausal | over 23 | 2b | None | None | WP+PAN+HDR-ICBT | 14 |
| D | Postmenopausal | over 23 | 3c2 | None | None | WP+PAN+HDR-ICBT | 24 |
| D | Premenopausal | under 23 | 1b1 | None | None | WP+PAN+HDR-ICBT | 53 |
| D | Postmenopausal | over 23 | 4a | Yes | None | WP+PAN+HDR-ICBT | 8 |
| D | Postmenopausal | under 23 | 3a | Yes | None | WP+PAN+HDR-ICBT | 28 |
| D | Premenopausal | over 23 | 3c1 | Yes | Yes | WP+PAN+HDR-ICBT | 52 |
| D | Postmenopausal | over 23 | 3c1 | Yes | Yes | WP+PAN+HDR-ICBT | 11 |
| D | Postmenopausal | over 23 | 3c1 | Yes | Yes | WP+PAN+HDR-ICBT | 8 |
| D | Postmenopausal | over 23 | 3c1 | Yes | Yes | WP+PAN+HDR-ICBT | 5 |
| D | Postmenopausal | over 23 | 4a | Yes | Yes | WP+PAN+HDR-ICBT | 11 |
| D | Postmenopausal | over 23 | 3b | Yes | Yes | WP+PAN+HDR-ICBT | 10 |
