# Supplementary material for: Incidence of pelvic fractures after definitive radiotherapy for cervical cancer: A retrospective multicenter cohort study (The IPFAR study)
Source: PLoS One. 2026 Mar 27;21(3):e0344384. doi: 10.1371/journal.pone.0344384 (PMC13028549; doi:10.1371/journal.pone.0344384)
Supplement: Attachment — Submitted filename: 20260206Response to Reviewers R1.docx [file pone.0344384.s003.docx]

Response to Reviewers

Manuscript ID: PONE-D-25-43659
Title: Incidence of pelvic fractures after definitive radiotherapy for cervical cancer: A retrospective multicenter cohort study (The IPFAR study)

Dear Academic Editor and Reviewers,

We sincerely thank the Academic Editor and the reviewers for their careful evaluation of our manuscript and for the constructive comments that have helped us improve the clarity and scientific rigor of the study. We have revised the manuscript accordingly and respond to each comment below. All revisions are reflected in the tracked-changes version of the manuscript.

Response to Academic Editor

Comment:
Issues regarding poor dosimetric data reporting and use of older treatment techniques should be addressed adequately along with limitations.

Response:
We thank the Academic Editor for this important comment. We agree that the absence of bone-specific dosimetric analysis and the predominant use of conventional radiotherapy techniques represent important limitations of this study. We have revised the Discussion and Conclusion sections to explicitly acknowledge these limitations and to avoid mechanistic interpretation of radiation dose effects. We also strengthened the description regarding the limited generalizability of our findings to modern IMRT/VMAT-based radiotherapy.

Reviewer #1

We sincerely thank Reviewer #1 for the positive and encouraging evaluation of our study and for recognizing the clinical relevance of the multicenter analysis.

No major changes were requested by Reviewer #1; however, the manuscript has been revised in response to Reviewer #2 and editorial comments to further improve clarity and interpretation.

Reviewer #2

We thank Reviewer #2 for the thorough and insightful review. We have carefully revised the manuscript to address all concerns raised.

Major Comment 1 — Lack of dosimetric bone analysis

Comment:
The manuscript implies a dose-effect relationship without reporting bone dosimetric parameters.

Response:
We agree with the reviewer that quantitative bone dosimetry would provide important mechanistic insight. Because this study was a retrospective multicenter investigation spanning multiple treatment planning systems and historical protocols, bone-specific dose–volume parameters were not consistently available across institutions.

We have therefore revised the manuscript to clarify that the absence of bone dosimetry precludes mechanistic interpretation. Statements suggesting dose-dependent effects have been rephrased to emphasize that our findings indicate only a spatial association between fracture location and irradiated regions. This limitation is now explicitly described in the Discussion.

Major Comment 2 — Treating institution as an independent risk factor

Comment:
Institution likely represents unmeasured confounders rather than a causal factor.

Response:
We agree with this important methodological point. We have revised the Discussion to clarify that treating institution likely reflects complex unmeasured variables rather than a direct causal determinant of fracture risk. We now explicitly discuss potential contributors such as differences in imaging surveillance intensity, imaging protocols, reporting thresholds for asymptomatic fractures, and subtle variations in treatment planning or delivery.

The interpretation of institutional variation has been revised to emphasize its hypothesis-generating nature.

Major Comment 3 — Absence of baseline bone mineral density

Comment:
Menopausal status is an imprecise surrogate for bone health.

Response:
We fully agree. Baseline bone mineral density data were not available in this retrospective dataset, which represents an important limitation. We have revised the Discussion to clarify that menopausal status should be interpreted as a surrogate marker for underlying bone health, and that the observed association may reflect residual confounding related to osteoporosis risk.

Major Comment 4 — Competing-risk framework

Comment:
Death and progression should be considered competing risks.

Response:
We agree that death and disease progression may function as competing risks for PIF occurrence. Because the primary objective of this study was exploratory, these events were treated as censoring. We have now added a statement in the Discussion acknowledging that competing-risk models could provide more precise estimation and should be considered in future studies.

Major Comment 5 — Applicability to modern radiotherapy

Comment:
Limited use of IMRT/VMAT restricts generalizability.

Response:
We agree and have strengthened this limitation in both the Discussion and Conclusion sections, emphasizing that most patients were treated using conventional four-field techniques with central shielding.

Minor Comments

Diagnostic criteria clarification

We added a concise standardized definition of PIF early in the Methods section.

Asymptomatic fracture inclusion

We clarified that both symptomatic and asymptomatic fractures detected on routine follow-up imaging were included.

Institutional tables

Tables were revised to clearly distinguish statistically compared variables from descriptive data.

Figures

Number-at-risk information and censoring indicators were added to survival figures.

Osteoporosis medication information

Medication information was clarified in tables and footnotes.

Speculative language

Statements regarding institutional quality control and technical nuances were revised to avoid speculative interpretation.

Language editing

The Discussion section was edited for clarity and conciseness.

Others

The order of the components in the Results section has been changed to make it easier to understand.

Journal Requirements

We have also addressed the journal’s technical requirements as follows:

- Supporting tables were incorporated into the main manuscript.
- The ethics statement now appears only in the Methods section.
- Supporting information files were corrected and re-uploaded.
- The author list was updated as requested.
- Table captions and supporting information captions were added.

Closing

We believe that these revisions have substantially strengthened the manuscript and addressed all reviewer concerns. We are grateful for the reviewers’ constructive feedback and hope that the revised manuscript is now suitable for publication in PLOS ONE.

Sincerely,

Shoji Nagao, MD, PhD
